# DEMENTIA-RELATED STIGMA, KNOWLEDGE, AND WILLINGNESS TO UNDERGO COGNITIVE SCREENING AMONG OLDER ADULTS

**DOI:** 10.1093/geroni/igad104.3266

**Published:** 2023-12-21

**Authors:** Whitney Kasenetz, Alex Crisci, Wendy Rote, Jennifer O’Brien

**Affiliations:** University of South Florida, St. Petersburg, Florida, United States; University of South Florida, St. Petersburg, Florida, United States; University of South Florida, St. Petersburg, Florida, United States; University of South Florida, Tampa, Florida, United States

## Abstract

Screening for dementia such as Alzheimer’s Disease may be most beneficial at the earliest detectable stages of disease presentation, yet there is no definitive recommendation for routine cognitive impairment screening and dementia remains underdiagnosed. Identifying perceptions of cognitive screening, stigmatized beliefs, and misconceptions about dementia could be beneficial for creating interventions that promote dementia knowledge to improve early screening and diagnosis and empower older adults to seek treatment sooner. Thus, we examined the extent to which attitudes, beliefs, and knowledge about cognitive screening and dementia predict willingness to undergo screening and subsequent screening behavior. 295 subjects aged ≥ 60 years completed online surveys containing the Perceptions Regarding Investigational Screening for Memory in Primary Care (PRISM-PC) questionnaire to assess attitudes about dementia screening and diagnosis, as well as the Alzheimer’s Disease Knowledge Scale (ADKS). Participants were then invited to complete the Montreal Cognitive Assessment in a university psychology laboratory. 94% of participants reported willingness to undergo screening, but only 69% completed one. Logistic regression analysis revealed that the screening acceptance scale of the PRISM-PC significantly predicted reported willingness to undergo screening but not the pursuit of screening. The other PRISM-PC subscales (perceived benefits of dementia screening, stigma of dementia screening, suffering from dementia screening, and impact of dementia screening on patient’s independence) and the ADKS did not predict willingness or pursuit of screening. While most were willing to undergo screening, significantly fewer completed screening within the study. The factors that predict pursuit of cognitive screening require further investigation.

